# Utility of Pediatric Nerve Biopsy in Tertiary Care Referral

**DOI:** 10.15844/pedneurbriefs-30-3-3

**Published:** 2016-03

**Authors:** Colin Quinn, Chafic Karam

**Affiliations:** 1 Department of Neurology, University of Pennsylvania, Philadelphia, PA; 2 Department of Neurology, Oregon Health and Sciences University, Portland, OR

**Keywords:** Biopsy, Diagnosis, Neuropathy, Pediatric, Peripheral Nerve

## Abstract

Investigators from Mayo Clinic in Rochester, MN, report the utility of nerve biopsy as a diagnostic tool in their institution.

Investigators from Mayo Clinic in Rochester, MN, report the utility of nerve biopsy as a diagnostic tool in their institution. The authors identified 316 nerve biopsies performed on patients <18 years of age between 1950 and 2009. Two thirds of these biopsies were performed within their institution, while the other third were performed externally, though all patients were evaluated at the Mayo Clinic and had clinically relevant details, electromyography (93%) and genetic testing results (14%). The utility of biopsies was determined by their effect on diagnosis (confirmation, change or refinement), treatment (with or clinical improvement) or additional testing. The authors found a distinct histopathologic diagnosis was present in 33% (106) of cases, with 33% (106) showing nonspecific histological abnormalities while the remaining biopsies were normal 29% (91) or could not be interpreted 5% (13). Clinical impact was credited to biopsy in 86% of cases (273). Two thirds of clinically impactful biopsies were attributable to refinement or change of the pre-biopsy diagnosis. Biopsy results altered treatment in 25% (80) of cases with clinical improvement seen in 18.6% (59). Additional testing was ordered based upon biopsy results in 20% (62%) of patients yielding significant diagnostic information in 8.8% (28) of patients. The reported nerve biopsy complication rate was 1% (3). [1]

COMMENTARY. This is a well conducted and important study looking at the role of nerve biopsy in a large series of pediatric patients. One limitation of the study is that it retrospectively evaluated the role of nerve biopsy from 1950 to 2009, a time when genetic testing was difficult. Today, some of these nerve biopsies may have been avoided. Another issue is that the study had a number of CIDP patients which in general do not require a nerve biopsy for diagnosis, since symptoms and signs as well as specific findings on nerve conduction studies and electromyography would establish the diagnosis. Despite these minor issues, there are 3 important messages to take from this study: 1) nerve biopsies in pediatric patients with normal nerve conduction studies are unnecessary 2) the role of nerve biopsy is still very important in specific situations and 3) the complications from the nerve biopsies are extremely low.

## References

[1] Ida CM, Dyck PJ, Dyck PJ, Engelstad JK, Wang W, Selcen D (2016). Pediatric Nerve Biopsy Diagnostic and Treatment Utility in Tertiary Care Referral. Pediatr Neurol.

